# Supplementary material for: Irbesartan May Ameliorate Ventricular Remodeling by Inhibiting CREB-Mediated Cardiac Aldosterone Synthesis in Rats with Myocardial Infarction
Source: Int J Mol Sci. 2024 Dec 29;26(1):198. doi: 10.3390/ijms26010198 (PMC11719508; doi:10.3390/ijms26010198)
Supplement: Supplementary file 1 [file ijms-26-00198-s001.zip › ijms-3324892-supplementary.pdf]

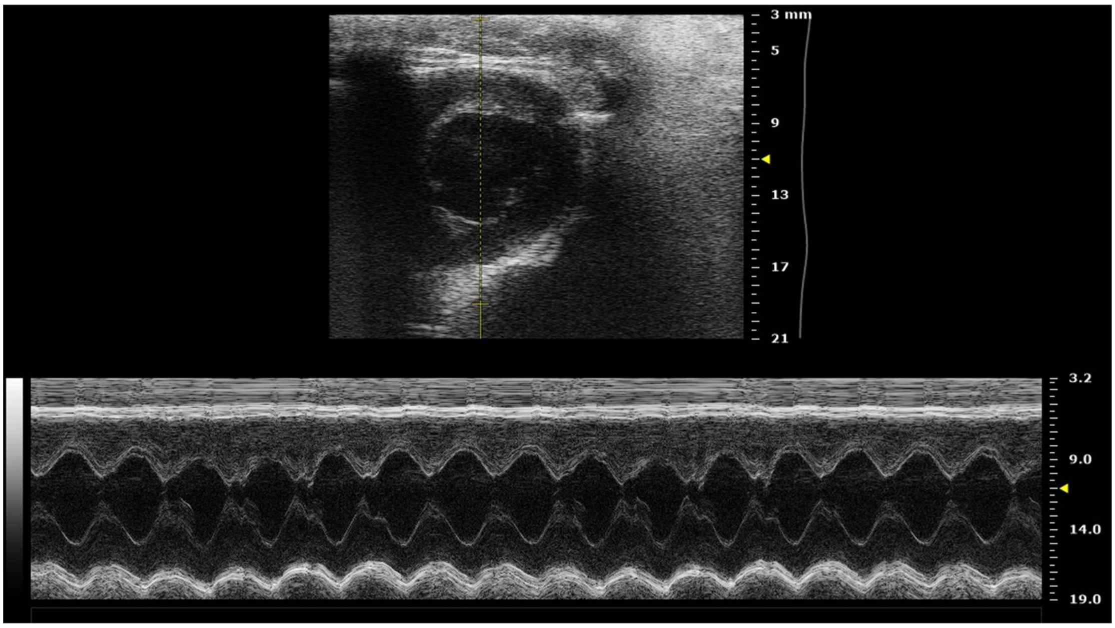

sham

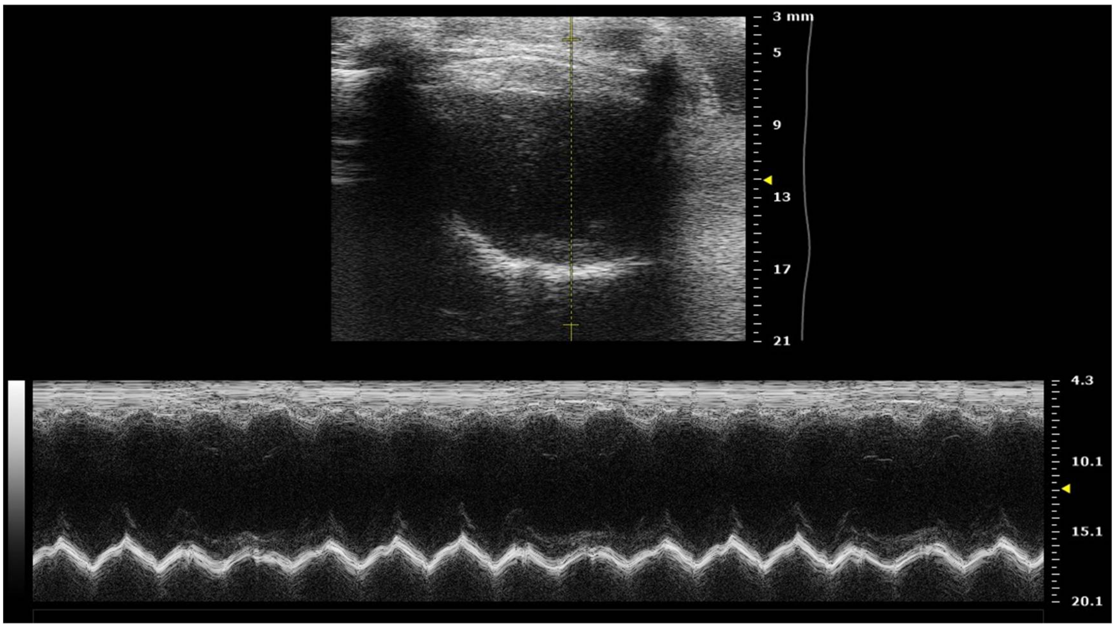

MI

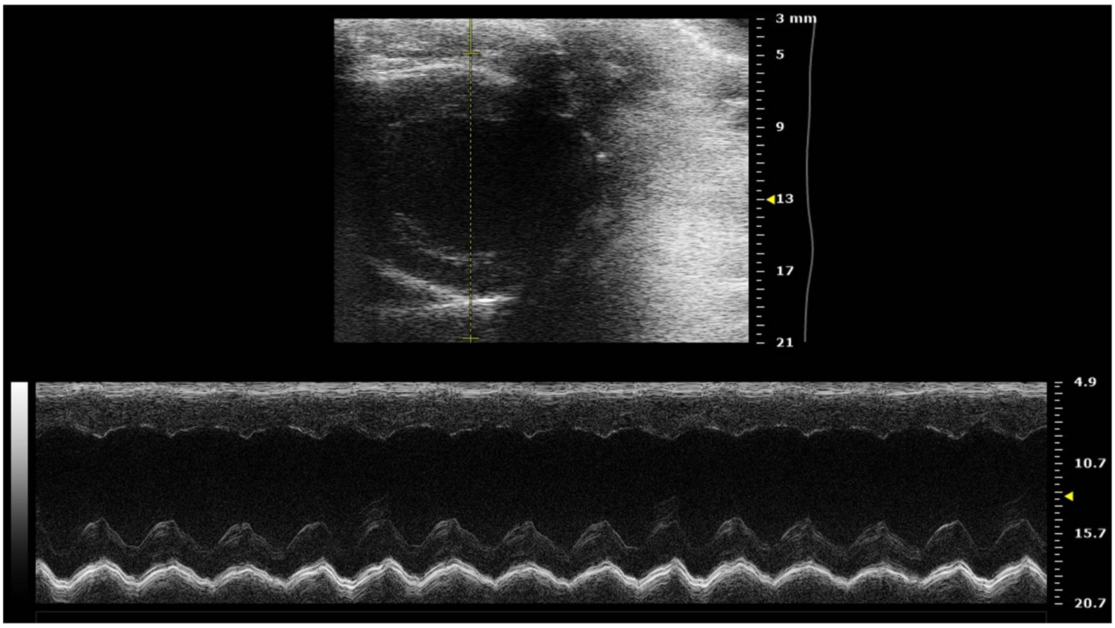

MI-irbesartan

Figure S1. Representative M-mode echocardiographic images of rats. sham, sham-operated rats; MI, myocardial infarction rats; MI-irbesartan, myocardial infarction rats fed with irbesartan.
